# Clinicopathological study of ameloblastoma and detection of human papilloma virus by immunohistochemistry

**DOI:** 10.12669/pjms.35.6.909

**Published:** 2019

**Authors:** Misbah Ali, Mulazim Hussain Bukhari, Faiza Hassan, Maria Illyas

**Affiliations:** 1 Misbah Ali, BDs, M.Phil. King Edward Medical University, Lahore, Pakistan; 2 Mulazim Hussain Bukhari, MBBS, DCP, CHPE, MPhil, FCPS, PhD. Head of Pathology Department, UCMD, University of Lahore, Lahore, Pakistan; 3 Faiza Hassan BDs, MPhil. Assistant Professor Oral Pathology, Fauji Foundation University, Islamabad, Pakistan; 4 Dr. Maria Illyas BDs, MPhil. Senior Registrar Oral Pathology, Baqai Medical and Dental College, Karachi, Pakistan

**Keywords:** Ameloblastoma, Human Papilloma virus, Immunohistochemistry, Odontogenic Tumors

## Abstract

**Objective:**

To study the clinocopathological factors and presence of Human Pappiloma Virus in ameloblastoma by immnohistochemistry.

**Methods:**

It was a cross sectional study on 50 surgical specimens of ameloblastoma, completed in six months. These were selected and processed for initial screening by H&E and then by immunohistochemistry (IHC) for detection of Human Papilloma Virus (HPV). The questionnaire was designed to study the clinicopathological factors associated in these patients. Sections of 4µm were cut, placed on special positive charged glass slides in the Department of Pathology, King Edward Medical University. It was then examined by the histopathologists for grading and scoring of these lesions. Chi Square test was used to assess the differences found in types of ameloblastomas. The p-value was smaller than 0.05 (p < 0.05).

**Results:**

The mean age of the patients (12-80 years old) was 38.6±15.1 years, with male-female ratio 2.84: 1. HPV was positive in 9 (18%), whereas negative in of 41 (82%) patients. Among the positive, reactive HPV with score-1 was 8 and score-2 was 1. According to histological variant, follicular was present in 78%, Plexiform pattern in 8%, Conventional and Desmoplastic variants in one patient each; and Cystic and Acanthomatous were seen in two and three patients respectively. The mandible was involved in 39 patients, maxilla and right maxilla involved in 4 patients each, right retromolar, cheek and angle of mandible was seen in one patient each. About 16% patients had anterior, 66% had posterior and 18% had both anterior and posterior regions involved. Among the HPV positive reactive statistically, no significant difference was found with smoking, Paan and exposure to pesticides, factory or mine (p-value > 0.05). Among HPV positive reactive patients, eight had ameloblastoma whereas, 1 had ameloblastomic fibroma. There was no statistical significance of type, location and region of tumor in HPV positivity.

**Conclusion:**

Mandible and posterior region was more commonly involved. Follicular pattern was most common. There was no effect of exposure to pesticides, factory or mine, smoke and human papilloma virus in the etiology of ameloblastoma because only 18% of patients showed the association of HPV16

## INTRODUCTION

Odontogenic tumours (OT) are benign tumors of the dental tissue arising from the mandible, the maxilla rarely from the gingiva. Broca first described an odontogenic neoplasm in 1868 and various classifications have been proposed.^1^ Ameloblastoma is the most common OT, approximately 1% of all oral tumours and affecting young adults in the fourth and fifth decades.^2^,^3^ As there was little clinical evidence for the classification into plexiform type and follicular type, the WHO subsequently reclassified ameloblastoma into solid/multicystic type, unicystic type, extraosseous/peripheral type, and desmoplastic type.^4^

Peripheral ameloblastoma and peripheral intraosseous ameloblastoma are two clinical variants of ameloblastoma, which present as an exophytic soft-tissue lesion.^5^ The pathogenesis of such tumors is still unclear. There are evidences of association of HPV with the development of benign and premalignant lesions, and malignant tumours. It has been found in healthy mucosa as well. HPV dominance in oral lesions, including oral cancers, has been shown to vary from 0% to 100%.^6^

The exposure to known risk factors such as use of tobacco, alcohol, betel quid and sexual intercourse may account for the variation. Now a day, HPV is considered as a sexually transmitted disease (STD).^7^ HPV has an important role in causing cervical carcinoma, with association of HPV16 and HPV18. The development of a successful HPV vaccine (GARDASIL) provided protection from HPV16 and HPV18 and is expected to reduce the need for surgery and global burden of cervical cancer throughout the world.^8^

Among different etiologic factors, HPV infection has been recently postulated to be somehow involved in the pathogenesis of ameloblastoma. Strong evidence has accumulated in the last 15 years showing that certain human papillomaviruses (HPVs) are etiologically involved in a subset of head and neck cancers (HNCs).^8^-^10^

Immunohistochemistry has emerged as the most valuable adjunct to routine H&E staining for accurate characterization of malignant neoplasms, especially in difficult and challenging cases. The accurate typing of malignant as well as benign tumors is important from diagnostic, prognostic, therapeutic, academic and research viewpoints, and immunohistochemistry is the main tool which has enabled the histopathologist to achieve this goal to know the clinicopathological factors and the presence of HPV.

## METHODS

The study design was cross-sectional and the duration of study was six months. The study was approved from IRB/Ethical Approval (No. 7774 REG/KEMU/2011) on 18.2.2010. The sampling was done by non-probability and purposive technique (Select the sample on the basis of our knowledge of the population and nature of our research aims). The sample size was calculated on the frequency of ameloblastoma, as it includes only 1% of the oral tumors, however, 50 samples were recommended by to the IRB to draw some inferential.^2^,^3^,^10^

**Fig. 1:**
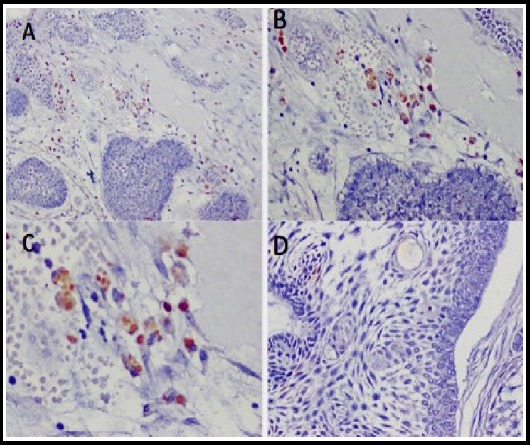
Microphotographs (A, B, & C) of ameloblastoma showing positivity in the nuclear areas for HPV-16, (IHC stain 10X, 20X and 40X magnification) respectively, while D, showing negative HPV on IHC stain.

The surgical specimens of ameloblastoma were selected from the oral and maxillofacial ward and processed for initial screening by hematoxylin and eosin. 2-3µm thin sections were taken on the slides, which were dewaxed in xylene and hydrated in alcohol. The slides were then rinsed in water, immersed in hematoxylin for 10 minutes and again washed till section became blue. Differentiation was done by immersing in 70% ethanol for 5-10 seconds and again washed for 10 minutes till sections were again blue. The slides were then stained in 1% eosin solution for 10 minutes and washed for 1-5minutes. Sections were then dipped in 95% and 100% ethanol and after drying, cleared with xylene and mounted in DPX (Fig. 1).

All the samples were fixed in 10% impartial formalin and inserted in paraffin. Sections of 4µm were cut, set on positively charged glass slides and immunohistochemical staining was performed on these specimens. For antigen recovery, the areas were submerged in 200 ml antigen recovery solutions, containing three drops of HCL, and warmed in microwave for 1 to 2 minutes. The tissue areas were cooled at room temperature for 1 hour.

With water, 5 minutes washing was given to these slides and then in another 5 minutes in PBS. These slides were then marked with pen. The primary antibody for HPV 16 & 18 was used for 24 hours. These slides were then washed in PBS washing was given for 5 minutes. The secondary antibody was used on these slides and these were Biotinylated goat (polycolonal) anti-mouse rabbit IgG (DAKO North America, Inc., Carpinteria, CA). The slides treated with these antibodies were incubated for 15-30 minutes at room temperature. These slides were rinsed with PBS for 5 minutes at pH 7.4 to 7.6. The concentrated Streptavidin peroxidase solution was poured on these slides and kept incubated for 30 minutes at 45ºC. After this procedure, washed with PBS for 5 minutes.^6^

The slides were smeared around the sections with undiluted substrate (3,3’-diaminobenzdine) containing one ml of DAB and one drop of Chromogenin. They were incubated for 3-5 minutes at room temperature taken after by flushing in PBS for 5 minutes. At the last, slides were counterstained with hematoxylin for 30 seconds and after that washed with tap water. After drying, slides were mounted with DPX.

The negative control comprised of the same tissue where only the PBS was used without primary antibody. It was at that examined under microscope by competent histopathologist. The case of cervical carcinoma with HPV 16 and 18 recognized on PCR was utilized as a positive control.

The association of smoking, paan and Pesticide, with HPV was also noted. The slides were studied by histopathologist and the IHC expression of HPV was seen in the nucleus of the cells. The data was entered in proforma and Chi square test was used to calculate the significant level for clinicopathological factors and presence of HPV in ameloblastoma. The significant level of p-value was smaller than 0.05 (p < 0.05).

## RESULTS

In this study, the mean age of the patients (12-80 years old) was 38.6±15.1 years. 8% patients were less than 20 years old; 56% were 21-40 years old; 26% patients were 41-60 years old and only 10% were 61-80 years old. A Significant Statistical difference (p=0.05) was seen youngers as compared to old age, middle age (Table-I).

**Table I:** Distribution of cases by age, gender and reactivity of HPV-16.

Age (Year) Mean age=38.6±15.1		Frequency	Percentage
< 20		04	08.0
21-40		28	56.0*
41-60		13	26.0
61-80		05	10.0
Total		50	100.0
***Gender*** M&F ratio 2.84: 1	Male	37	74.0
	Female	13	26.0
Positive for HPV		09	18.0
Negative for HPV		41	82.0
Mean±SD		38.6±15.1	

* Significant Statistical difference was seen youngers as compared to old age, middle age and children (p<0.05). A Significant Statistical difference was seen between males and females (p<, 0.05). No significant association of HPV-16 was seen in Ameloblastoma any variant however a significant difference was seen with non reactivity of HPV, Ameloblastoma (p>0.05; chi-square = 41.0, degrees of freedom = 1 and probability = 0.000), The presence of HPV may n=be may accounted but some other some other factors may be investigated for the association with ameloblastoma

There were 37 males and 13 females with male-female ratio 2.84: 1. HPV was positive in 9 (18%), whereas negative in of 41 (82%) patients. Among the positive, reactive HPV with score-1 was 8 and score-2 was 1 (Table-I).

According to histological variant, follicular was present in 78%, Plexiform pattern in 8%, Conventional and Desmoplastic variants in 1 patient each; and Cystic and Acanthomatous were seen in 2 and 3 patients respectively. According to the location of the tumor, mandible was involved in in 39 patients, maxilla and right maxilla involved in 4 patients each, right retromolar, cheek and angle of mandible was seen in one patient each. The 16% patients had anterior, 66% had posterior and 18% had both anterior and posterior regions involved. (Table-II)

**Table II:** Histological variant of Ameloblastoma.

Histological variant	Number	Percentage
Follicular*	39	78.0
Plexiform pattern	04	08.0
Conventional	01	02.0
Cystic	02	04.0
Acanthomatous	03	06.0
Desmoplastic	01	02.0

Total	50	100.0

A Significant Statistical difference was seen between Follicular variant and others (p<0.05); chi-square = 31.4 degrees of freedom = 1 and probability = 0.000).

In this study only 9 (18%) patients were smokers and 41 (82%) were non-smokers. The 45 patients were not exposed to pesticides, whereas 10% patients were exposed. The 18% patients were habitual of paan, while 82% didn’t have such habits. (Table-III)

**Table III:** Smoking habit.

Smoking		Total
***Yes***	***No***
09 (18%)	41 (82%)	50
***Pesticides***		
05 (10%)	45 (90)	50
***Habit of Paan***		
09 (18%)	41 (82%)	50

No association of smoking, Paan or pesticides for any variant Ameloblastoma (p>0.05; chi-square = 41.0, degrees of freedom = 1 and probability = 0.000), the possibility may be accounted but some other some other factors may be investigated for the association with ameloblastoma

Among the HPV positive reactive, 6 were males and 3 were females with insignificant difference of gender (p-value > 0.05). No patient was smoker; one was habitual of Paan and no patient not exposed to pesticide and factory or mine. Statistically, no significant difference was found with smoking, Paan and exposure to pesticides, factory or mine (p-value > 0.05). Among HPV positive reactive patients, 8 had ameloblastoma whereas, one had ameloblastomic fibroma. There was no statistical significance of type, location and region of tumor in HPV positivity (Table-III).

## DISCUSSION

Ameloblastoma is regarded as the most clinically significant tumor of odontogenic origin since it is locally aggressive and has a very high recurrence rate after inadequate or conservative treatment.^2^,^3^ It constitute approximately 1% of all oral tumors and about 12% of odontogenic tumors. Human papilloma viruses (HPV) are DNA viruses that infect squamous epithelium at selected locations in skin and mucosa. These viruses induce papillomatous, hyperplastic or verrucous lesions.^11^,^12^

The purpose of this study was to investigate the typical histological changes caused by human papilloma virus in ameloblastoma by immunohistochemistry. The data collected in this study provides clinicopathological information which is of significance to the pathologists and clinicians. The mean age of the patients with ameloblastoma in our study was 38.6±15.1 years; which is in coincidence with the studies, conducted by Singh et al. in 2010^13^; Morbini et al. in 2019^16^, Adeline et al. in 2008^15^ where mean ages were reported 38.3 years, 38 years and 30.2 years respectively. However, results showed a marked variation from study by Kahn MA in 1999^15^, in which the mean age was 10.4 years. This variation may be due to the late presentation and lack of awareness of the patient.

The male to female ratio in present study was similar to the study conducted by Singh et al. in 2010^13^, whereas in contrast to results of some other studies^16^ with male-female ratio of 1:1. This variation may be due to the difference in the sample size as author reviewed the literature of amelobalastoma from 1963 to 1993. Only 5(10%) of 50 patients of ameloblastoma in the present study were exposed to pesticides and 45 (90%) of 50 patients were not exposed. One of the 50 patients was exposed to factory or mine and 49(98%) had never visited to factory or mine. No significant information was found about the role of pesticides and exposure to factory or mine in ameloblastoma in literature. The findings are consistent with the Rubini et al 2017.^17^

Location of the ameloblastoma in the present study showed that 42 cases involved mandible and 18 cases involved maxilla. These findings were similar to the study of Singh et al. (2010)^13^ in which they reported 95% of tumor occurring in mandible and 5 % in the maxilla. Mehngi et al. in 2016^18^ also observed that 89% of cases were in mandible and 11% were in maxilla. The results were also similar with studies conducted by Adeline et al. 2008^15^; Ledesma-Montes et al., 2007^19^, Odukoya et al. in 2008^20^; and Lee et al. 2013.^21^

In our study only 9 patients 18% patients were habitual of betel nut chewing, and smoking while rest of 41 (82%) patients did not have such habits. Regarding pesticides, only five patients (10) patients were exposed to this while 90% patients with this tumor did not give the history of exposure. In literature no information is available about the impact of betel nut chewing in ameloblastoma. No association of smoking, Paan or pesticides for any variant Ameloblastoma (p>0.05; chi-square = 41.0, degrees of freedom = 1 and probability = 0.000), it means some other factors may be investigated for the association with ameloblastoma.

The most common histological type of ameloblastoma in the present study was of follicular variant. 78% cases were of follicular type; 8% were of plexiform pattern and only 2% was of desmoplastic type. Cystic and acanthomatous variants were seen in 4% and 6% respectively. Fulco et al. in 2010^22^ also found follicular as the most common variant of ameloblastoma. They found 77.6% follicular, 69.4% acanthomatous, 65.3% plexiform and 22.4% desmoplastic ameloblastoma. A Significant Statistical difference was seen between Follicular variant and others (p<0.05); chi-square = 31.4, degrees of freedom = 1 and probability = 0.000.

The present study showed that the posterior region of mandible was involved most commonly than anterior region. There were 66% cases located in the posterior region of mandible, while 16% cases were found in anterior region and in 18% occupied both the anterior and posterior region. These results agree with those studied by Singh et al. in 2016^23^ and Reichart et al. in 1995^24^, who found that posterior region of mandible is most commonly involved. In the present study only 18% patients were smokers and 82% were non smokers. This study agrees with that of Guan et al 2019.^25^

In our study Human papilloma virus (HPV) was detected by immunohistochemistry in 18% samples of ameloblastoma and in rest of 41 samples HPV was negative. Tang X in 2005^26^ also found HPV in 9 out of 20 samples of ameloblastoma. However, these results were in contrast from those found by Correnti M et al. in 2011^27^ who found that all cases of ameloblastoma were HPV negative when detected by immunohistochemistry. This may be due to the small sample size taken by authors in their research. Among the positive HPV cases in our study, 16% cases were score one and only one case was of score two. No data is available about the scoring of HPV in ameloblastoma in literature.

### Limitations of the study

This study cannot suggest that Human papilloma virus is a risk factor for ameloblastoma. Thus the present study can be further evaluated with a bigger sample size and Human papilloma virus can be detected with a more sensitive technique like Polymerase chain reaction (PCR).

## CONCLUSION

HPV positivity was detected in few cases of ameloblastomas HPV positivity was observed more in the follicular cases than another type. Ameloblastoma was more common in Mandible and posterior region was more commonly involved. There was no effect of exposure to pesticides, factory or mine, smoke and human papilloma virus in the etiology of ameloblastoma.

## RECOMMENDATION

The diagnosis of any tumor either malignant or benign depends on its histopathology. Good histopathological findings lead to correct diagnosis. Clinicopathological study and etiology of any tumor play an important role in its treatment. With detailed study of clinical and histopathologic findings, keeping in mind the treatment outcome, the possible HPV etiology, may be included. The preliminary nature of finding high risk or low risk HPV in the ameloblastoma is stressed, with recommendation for further verification and typing with the more sensitive *in situ* hybridization technique.

This requires a detailed knowledge of all the clinical and pathological factors. This study involves various clinicopathological factors and the role of human papilloma virus in the etiology of ameloblastoma.

### Authors’ Contributions:

**MA,** conceived and design of the work, collected, analyzed the data with interpretation.

**MHB,** supervised the work, played main role in drafting the article, Critical revision of the article and gave Final approval of the version to be published.

**FH,** helped in analyzing the data.

**MI** helped in interpreting the data.
